# Fatal *Acinetobacter baumanii* Necrotizing Fasciitis following Open Reduction Internal Fixation in a Polytrauma Patient

**DOI:** 10.1155/2018/4176320

**Published:** 2018-06-21

**Authors:** Alexandre Nehme, Nabih I. Joukhadar, Elias Saidy, Mohammad Darwiche, Dany K. Aouad, Hicham G. Abdel Nour

**Affiliations:** Department of Orthopedic Surgery and Traumatology, Saint Georges University Medical Center, Balamand University, P.O. Box 166378, Achrafieh, Beirut 1100 2807, Lebanon

## Abstract

Necrotizing fasciitis is an uncommon and potentially fatal infection that can affect the epidermis, dermis, and more commonly the subcutaneous, fascia, and muscle layers. NF is usually caused by toxin-producing bacteria with a relatively fast progression associated with severe surrounding tissue destruction. Early diagnosis and management are crucial factors for survival. Broad-spectrum antibiotics along with surgical debridement, sometimes multiple, are needed in order to stop or slow down the progression of NF. Despite optimal care, necrotizing fasciitis remains a highly morbid condition with a high mortality rate. We present a case of a 28-year-old male patient with rapidly developing fatal *A. baumannii* associated with necrotizing fasciitis, after open reduction and internal fixation (ORIF) of multiple fractures after polytrauma.

## 1. Introduction

Necrotizing fasciitis (NF) is a rare, life-threatening bacterial infection leading to necrosis of the fascia, underlying skin, subcutaneous tissue, and vasculature associated with high morbidity and mortality. Necrotizing fasciitis could affect the perineum, the genital area, and most commonly the lower extremities [1–3], with a male-to-female ratio of 3 : 1 in terms of predominance [1].

It is a rapidly progressing condition, making timely clinical diagnosis challenging and taking immediate measures the most important factors for survival and treatment. Some predisposing conditions are diabetes mellitus, alcoholism, hypertension, and malignancies. In addition to these conditions, obesity, chronic kidney dysfunction, and lymphocytic dysfunction leading to immunosuppression are all predisposing factors to NF [4–6].

NF is often misdiagnosed as cellulitis or other similar skin and soft tissue infections such as erysipelas [1].

It is a polymicrobial infection in approximately 70% of cases. The most common pathogens associated with necrotizing fasciitis include group A beta-hemolytic *Streptococcus*, group B *Streptococcus*, Enterococci, coagulase-negative Staphylococci, *Staphylococcus aureus*, *Escherichia coli*, *Pseudomonas aeruginosa*, *Bacteroides*, and *Clostridium* [7]. *Acinetobacter baumannii* is rarely associated with necrotizing fasciitis, especially in immunocompetent hosts [8].

We report an unusual case of a male patient with rapidly developing fatal *A. baumannii* associated with necrotizing fasciitis, after open reduction and internal fixation (ORIF) of multiple fractures after polytrauma.

## 2. Case Report

This is a case of a 28-year-old male patient, previously healthy, with no past surgical history. He was transferred to our facility from a peripheral hospital due to multiple fractures and crush injuries sustained after a motor vehicle accident one week prior to presentation.

The patient had a butterfly fracture at the junction of the proximal and middle thirds of the left femur (already treated by ORIF using a long DCS plate), a left olecranon fracture (already treated by ORIF using tension band wiring), a right leg compartment syndrome (already treated by partial fasciotomy), a right calcaneal fracture (treated conservatively using a cast), a nondisplaced maxillary fracture, and a nondisplaced T12 vertebral body fracture.

Doppler sonography done prior to presentation revealed weak flow in the posterior tibial artery and absence of dorsalis pedis flux in the right lower extremity with normal flow in the left lower extremity. The patient was suffering from acute kidney injury and on daily dialysis (creatinine level on presentation was 6.04 mg/dl; normal range in adult males is 0.6–1.2 mg/dl), attributed to myoglobinuria caused by severe rhabdomyolysis (CPK : 115,000 U/L;normal range: 22–198 U/L).

**Figure 1:**
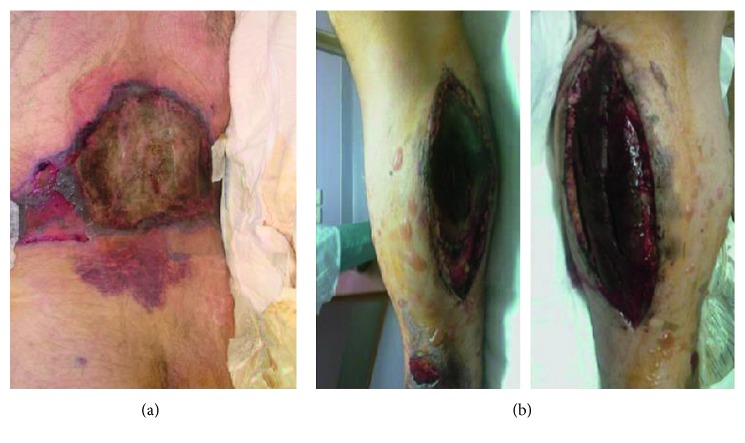
(a) Mid-lower back laceration, nonpurulent upon presentation with minimal discharge. (b) Right leg open incision sites after fasciotomy due to the right leg compartment syndrome after trauma.

Physical examination at presentation revealed an awake, oriented, and cooperative patient with several skin lacerations in the right lower extremity and mid-lower back (Figure 1(a)), right leg open incision sites after fasciotomy (Figure 1(b)), a left thigh lateral incision site, and a left elbow posterior incision site after ORIF. There were signs of right eye infection (blepharatis) and right tibial wound infection suspected by erythema and purulent discharge.

The laboratory tests done upon arrival revealed a C-reactive protein level of 7 mg/dl (normal range: 0-1.0 mg/dl) and a high WBC count 35 × 10^9^/L (normal range: 4.00–11.0 × 10^9^/L), suggesting a possible deep infection. On presentation, he was already receiving amoxicillin and metronidazole started at another facility for an unjustified reason.

On presentation, the patient was suffering from severe right leg pain associated with right foot paresthesia and pain upon passive and active range of motion. He was diagnosed with right lower extremity compartment syndrome necessitating an immediate extension of the right fasciotomies to the level of the ankle. For broader coverage, the patient was placed on imipenem, vancomycin, and nystatin by the infectious disease team awaiting cultures from blood, right eye, and right tibial wound.

Two days after presentation, the tibial wound culture grew *Acinetobacter baumanii* that was sensitive to imipenem.

Daily hemodialysis sessions and daily wound care was performed. An inferior vena cava filter was placed. A right internal jugular central catheter was also placed to allow adequate perfusion.

Extensive daily dressing changes and debridement were done to his right lower extremity wound. On day 4, no significant improvement was noted despite wound care on antibiotic therapy. The right tibial wound was found to be diffusely necrotic with irreversibility of drainage, and the patient started manifesting secondary systemic complications. He was therefore transferred to the Operating Room (OR) for exploration and a possible amputation at the level of the affected lower limb. A circumferential incision was made at the level of the proximal 1/3 of the right tibia, and muscles explored were found to be fully necrotic and nonviable with purulent secretions suggesting continuing active infection of the deep tissues. The best course of action determined was below the knee amputation, in an attempt to salvage as much of the proximal tissues as possible, giving them the chance to heal and recover while monitoring. Given the necessity and the importance of the procedure, it was performed on the spot in the OR.

The culture of the amputation stump grew extended-spectrum beta-lactamase (ESBL) producing *Escherichia coli* and *Acinetobacter baumannii*.

**Figure 2:**
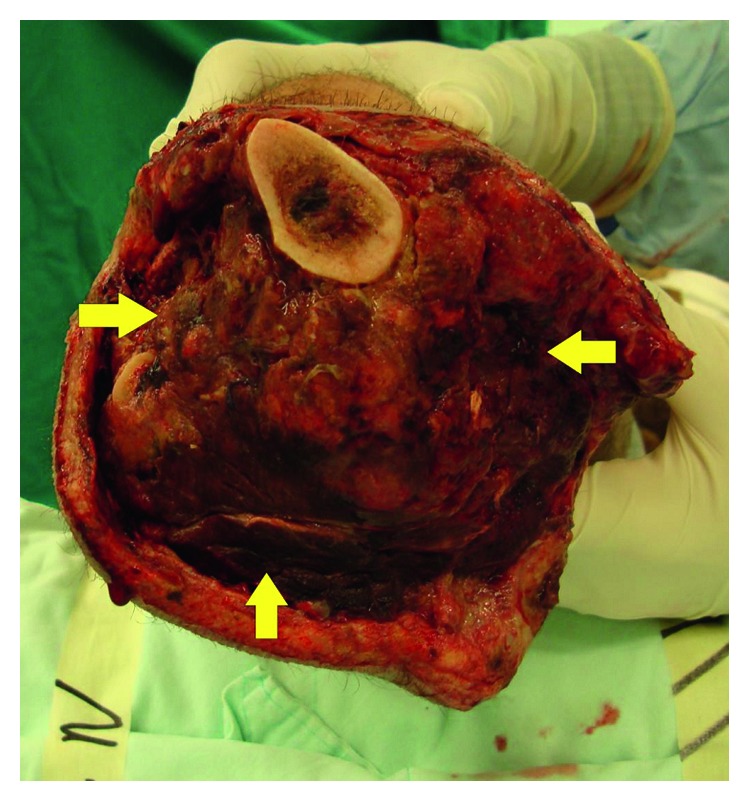
Progression of necrosis at the amputation stump, requiring further debridement of the necrotic edges.

The patient's systemic overall deterioration with severe anemia (Hg = 6.4 g/dl), persistent acute renal failure, and necrosis of the edges of the right amputation stump dictated a transfer of the patient to the OR on day 8 after admission and debridement of the necrotic edges of the right amputation stump (Figure 2). However, this intervention did not halt the progression of the ongoing myonecrosis at the amputation site.

**Figure 3:**
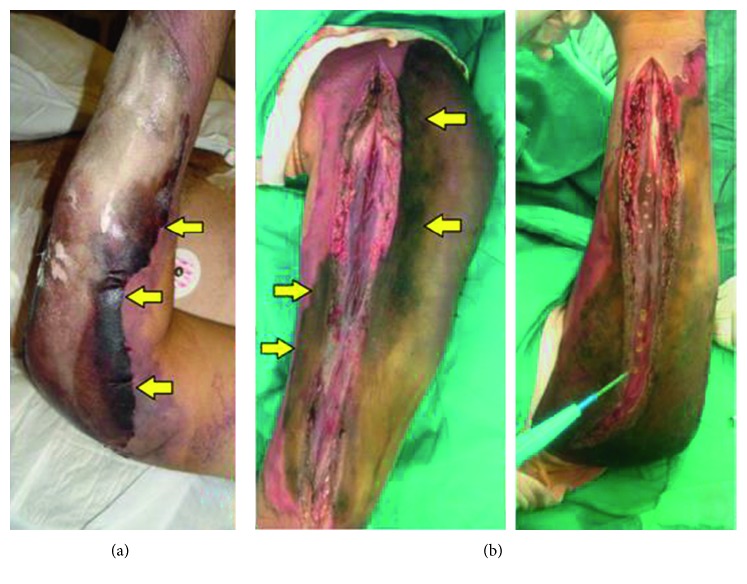
(a) Signs of infection with purulent discharge and progressive expanding necrosis on the left elbow suture line. (b) Posterior and anterior compartment fasciotomies, with inspection of deep necrotic tissues extending from the skin down to the muscles. No twitching seen after cauterization stimuli testing.

On day 9 of his hospitalization, the left elbow suture line started showing signs of wound infection and progressively deteriorated showing necrotic tissue (Figure 3(a)). On day 13, the patient was taken to the OR for evaluation under local anesthesia. Needle puncturing of the arm, forearm, and fingers were performed and did not show the presence of blood. The decision was taken to perform anterior and posterior skin release and bicompartmental fasciotomies of the forearm (Figure 3(b)). All explored tissues (skin, subcutaneous tissue, and muscles) were found to be necrotic, with no twitching after cauterization stimuli testing. Inspection showed necrotic, tense, and circumferential 3rd degree burnt skin, 10 cm both proximal and distal to the left elbow wound. Examination revealed very weak radial pulse and cyanotic finger tips. The right BKA amputation stump still showed necrotic tissues so debridement till acceptable perfusion was performed. The left thigh wound was explored, and slight debridement was carried out.

**Figure 4:**
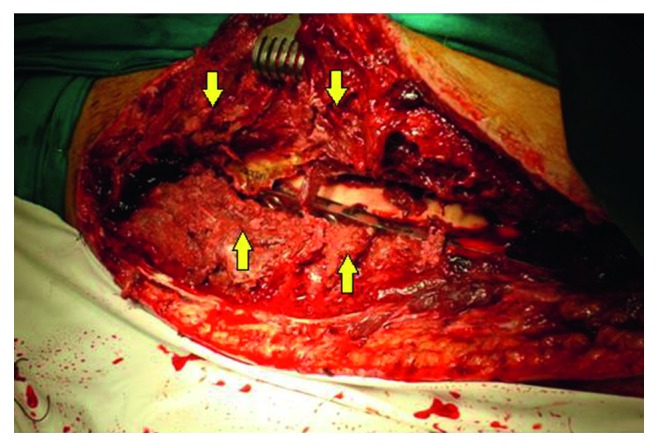
Left thigh incision site found to be necrotic with diffuse pyomyositis extending to the anterior and posterior compartment, which lead to performing an aggressive debridement and excision of necrotic tissues that reached the bone.

After reviewing the facts at hand, due to the development of systemic toxicosis in the context of several infected myonecrotic wounds in nonviable limbs, and with no improvement in renal function, the decision for extensive debridement under general anesthesia was made. On day 14 of presentation, the patient was taken to the OR for reevaluation, exploration, and possible management of his progressively necrotic left thigh incision site. Incision and drainage revealed diffuse pyomyositis extending to the anterior and posterior compartment, which lead to performing an aggressive debridement and excision of necrotic tissues that reached the bone (Figure 4).

The left upper extremity was reevaluated: due to the extensive necrosis extending from the fingers to the forearm and diffuse pyomyositis reaching up to 8 cm proximal to the elbow, the decision to perform a transhumeral amputation was made.

The right lower extremity amputated necrotic stump was reevaluated, showing diffuse necrosis and pyomyositis, resulting in the extension of the amputation to above the knee.

The lesion located in his lower back at the level of the lumbosacral spine was explored, where a 20 × 30 cm necrotic skin patch was incised. Deep necrotic lower back paraspinal muscles were also debrided. The debridement was stopped since there was a high risk of retroperitoneal breach at the level of the flanks.

The diagnosis of advanced unresponsive necrotizing myositis with poor prognosis and low survival rate was communicated to the family immediately post-operatively. The patient was transferred intubated from the OR to the ICU for adequate monitoring and critical management.

On the night of day 15 after presentation, the patient developed refractory septic shock due to extensive necrotizing myositis and infected wounds by *Acinetobacter baumanii*. These were complicated shortly after, by asystole that did not respond to full adult advanced cardiac medical and mechanical reanimation for around 45 minutes leading to the patient's death.

## 3. Discussion

NF is an exceedingly rare clinical entity, with an estimated 1000 cases annually in the United States with an increase of 35% in hospitalizations between 2001 and 2010 [1].

However, it appears that this incidence has been increasing. The cause for this increase is unclear, but it may be a result of greater awareness of the problem leading to higher rates of reporting, increasing bacterial virulence, increased antimicrobial resistance, or all three [9]. Most commonly, NF is classified on the basis of microbiology as type I, being polymicrobial and the most common type accounting for 70% to 90% of the cases [1]. Type II, being monomicrobial and is usually caused by group A streptococcus and *Staphylococcus aureus* [1]. Type III, being caused by marine *Vibrio* species and *Clostridium* species, is the most rapidly progressing deterioration with a high chance of multiorgan failure within 24 hours [1]. Type IV most often is caused by fungal infections in immunocompromised hosts [1].

These classification systems are not clinically significant as they do not change the management of the patient [10].

Clinically, it is characterized by a rapidly progressing necrosis of both the fascia and subcutaneous tissue. The fulminant tissue necrosis may rapidly progress into septic shock with multiorgan failure [11]. Once symptomatic, the progression of disease is typically measured in hours; early diagnosis and treatment are crucial to survival [9]. It is a surgical emergency with an associated mortality as high as 75%. When NF is established or highly suspected, urgent exploration and debridement of tissue is the cornerstone of obtaining a diagnosis and successful management. During surgery, the findings of grayish necrotic fascia, decrease in resistance to blunt dissection, absence of bleeding of the fascia, and presence of foul-smelling purulent discharge are diagnostic [10]. Debridement of the necrotic tissue should be undertaken as soon as possible. Other researchers have clearly shown the impact of early and complete debridement on final outcome in patients with NF [7, 12].

*A. baumanii*, an aerobic, Gram-negative bacillus, ubiquitously isolated from soil, water, and healthcare settings, is increasing in prevalence [1].

This Gram-negative bacillus is being increasingly implicated in nosocomial infections. This bacterium is known to cause ventilator-associated pneumonia, blood infections, wound infections, and catheter-related infections [9]. This pathogen produces AmpC cephalosporinases and aminoglycoside modifying enzymes with an ability to upregulate its efflux pumps and downregulate its outer membrane porins. Moreover, this organism has been known to have a heightened ability to acquire resistance from the environment [11]. Up to 30% of *A. baumanii* isolates from intensive care units (ICUs) have been shown to be multidrug resistant (MDR) and have been associated with increased mortality rates [10].

Increasing case studies from all around the world have been reporting *A. baumannii* as the cause of soft tissue infections [7, 8, 12–14]. In all these cases, the *A. baumannii* infection progressed and caused sepsis. In a study, 4.5% of blood cultures taken from patients hospitalized in all departments were identified as *A. baumannii* infections [15]. In neonates, 10.8% of blood isolates were identified as *A. baumannii* and were implicated with 11.3% mortality rates [15].

Reviewing the clinical history and culture results since his admission, the patient had several wound and soft tissue infections, most probably nosocomial in nature either acquired from our hospital or from the previously visited facility. In this context, multidrug-resistant *A. baumanii* has been constantly isolated from the affected eye, right tibia wound (before and after amputation), and his left upper extremity wound. It is clear that the treatment could not contain the infection and was not successful in eradicating the organism that was subsequently isolated from left thigh previous incision site. One day later, the same organism was isolated from the sputum of the patient shortly before he developed the septic shock. The presence of other organisms such as *E. coli* and *Enterobacter* spp. contributed to the severity of the case, especially, *E. coli* that was isolated from the right tibia and the muscle biopsy intraoperatively. However, the organism that was constantly found in all cultures was MDR *A. baumanii* and was therefore incriminated as the major cause for the generalized infections.

## 4. Conclusion

In conclusion, necrotizing fasciitis is a rare life-threatening condition that requires prompt recognition and aggressive surgical debridement along with broad-spectrum antibiotics. Early diagnosis of the condition poses a challenge, and a high index of suspicion is crucial.

The question at hand remains. Would a more aggressive and a faster decision to intervene have affected the outcome in this patient or was he doomed since the beginning of the deep tissue infection? And, are there any promising alternatives for the treatment of necrotizing fasciitis or improvements on the current standard of care consisting of antibiotics and surgical debridement?
